# Unnecessary ordering of magnetic resonance imaging of the knee: A retrospective chart review of referrals to orthopedic surgeons

**DOI:** 10.1371/journal.pone.0241645

**Published:** 2020-11-02

**Authors:** Heba Tallah Mohammed, Samuel Yoon, Thomas Hupel, Lori-Anne Payson

**Affiliations:** 1 eHealth Centre of Excellence, Kitchener, Ontario, Canada; 2 Division of Orthopaedic Surgery, University of Toronto, Toronto, Ontario, Canada; 3 Department of Surgery, McMaster University, Hamilton, Ontario, Canada; 4 Department of Family Medicine, McMaster University, Hamilton, Ontario, Canada; Humanitas Clinical and Research Center - IRRCS, ITALY

## Abstract

There is a noticeable increase in the unnecessary ordering of Magnetic Resonance Imaging (MRI) of the knee in older patients. This quality improvement study assessed the frequency of unnecessary pre-consultation knee MRIs and investigated the effect on the outcome of the patients’ consultation with the orthopedic surgeon. 650 medical charts of patients aged 55 years or older referred to an orthopedic clinic with knee complaints were reviewed. Patients arriving with a pre-consultation MRI were identified, and the usefulness of the MRI was evaluated using the appropriateness criteria developed to support this study. Of the 650 patient charts reviewed, 225 patients presented with a pre-consultation MRI, 76% of which were not useful for the orthopedic surgeon. The ordered knee MRI scans were considered not useful because they were requested for confirmed meniscal tear for patients ≥55 years, suspected degenerative disorder and ligament/tendon injury, or for patients with severe osteoarthritis without locking or extension. These MRI scans were done despite the absence of signs of effusion, tenderness, soft tissue swelling, decreased range of motion, or difficulty of weight-bearing, a lack of persistent knee joint pain at the time of assessment, or with no x-ray before ordering MRI. Half of the patients with a pre-consult MRI did not present with plain radiographs of their knee, however, 35% of those still required an x-ray to be ordered at the time of the surgical consult. A logistic regression analysis on post-consult disposition found that patients with pre-consult MRI were less likely to be considered for total knee arthroplasty (TKA) (OR 0.424, CI 0.258–0.698, p = 0.001). Patients assessed by an advanced practice physiotherapist prior to referral for surgical consult were 4.47 more likely to have TKA (CI 2.844–7.039, p< 0.000). Most of the pre-consult knee MRIs were deemed as unnecessary for the orthopedic surgeon’s clinical decision-making. This study highlights the potential benefits of following a comprehensive model of care within the referral process to reduce the unnecessary high orders of pre-consult MRI scans.

## Introduction

Magnetic resonance imaging (MRI) allows for a non-invasive, detailed examination of musculoskeletal derangements [1]. The volume of MRIs ordered to guide clinical diagnoses has continued to increase in Canada rapidly [1]. In 2010, Canadians underwent 1.4 million MRI tests [2]. In Ontario alone, an increase in the number of MRI scans from 10 to 60 per 1000 people has been documented between 2000 and 2016 [3]. Over 80% of all Canadian MRI tests for outpatients are of the head, spine, and extremities [2]. According to the office of the Auditor-General Ontario (2018), MRI scans have increased by 17% over the past five years. Currently, MRI scans of the extremities alone account for 20% of the total MRI scans in the province of Ontario [4]. While there is a legitimate, demand-based growth [2, 5], there is also a noticeable increase up to 30% in unnecessary MRI ordering [3, 6].

Currently, knee MRI examinations are a cause of controversy as they are frequently requested and often not indicated [6]. MRI is a sensitive tool for identifying soft tissue pathologies; however, it is of low specificity in determining significant clinical lesions in the knee [7]. This is particularly relevant for older patients with knee pain due to the high prevalence of degenerative changes [8, 9]. A full comprehensive history, thorough physical examination, and radiographic findings are fundamental factors in evaluating patients with arthritis and determining its stage for an informed decision on the management plan [1, 5, 7].

The high rate of ordering MRI before orthopedic consultation is influenced by many factors that include, but not limited to, the pressure that patients place on primary care physicians (PCPs) for timely access to services and the long wait time for orthopedic patients to access care [2, 10, 11]. Furthermore, the recent advancement in the diagnostic imaging (DI) technology, and direct access make MRI a more common way to obtain information, the gap in PCPs’ level of experience and training in evaluating orthopedic patients [2, 10], and the diversity in the utilization of evidence-based practice guidelines when ordering DI [1,12–14].

The goal of this quality improvement retrospective chart review study was to assess the frequency of MRI scans ordered prior to the orthopedic consult, determine the volume deemed to be clinically unnecessary and to document the reasons for the MRI ordered. The sample was narrowed to only include patients over the age of 55 with knee pain who were assessed at an orthopedic clinic in Kitchener, Ontario. This study also aimed to investigate the association between the ordering of pre-consult MRIs and the outcome of the surgical consultation.

## Materials and methods

A retrospective chart review was conducted to collect data on patients referred to an orthopedic clinic in Kitchener- Waterloo for a knee pain consult over a period of one-year from October 2016. One of the coauthors (SY), an orthopedic resident—with a year of training experience in orthopedics at the time of data collection- without a clinical role at the participating orthopedic practice, reviewed all patient charts. The orthopedic resident was mentored and supervised by one of the coauthors (TH)- an orthopedic surgeon. The resident identified patients 55 years or older who were referred for chronic knee pain with no history of tumour, cancer or previous knee surgery to be included in the chart review. Patients who did not meet the inclusion criteria were excluded from the chart review. The patient data was extracted from referral and consultation documents, the electronic medical record, as well as diagnostic imaging reports. All patient information was anonymized for analysis and kept in a password protected Microsoft Excel file.

### Determination of unnecessary ordering of MRI imaging

**Fig 1:**
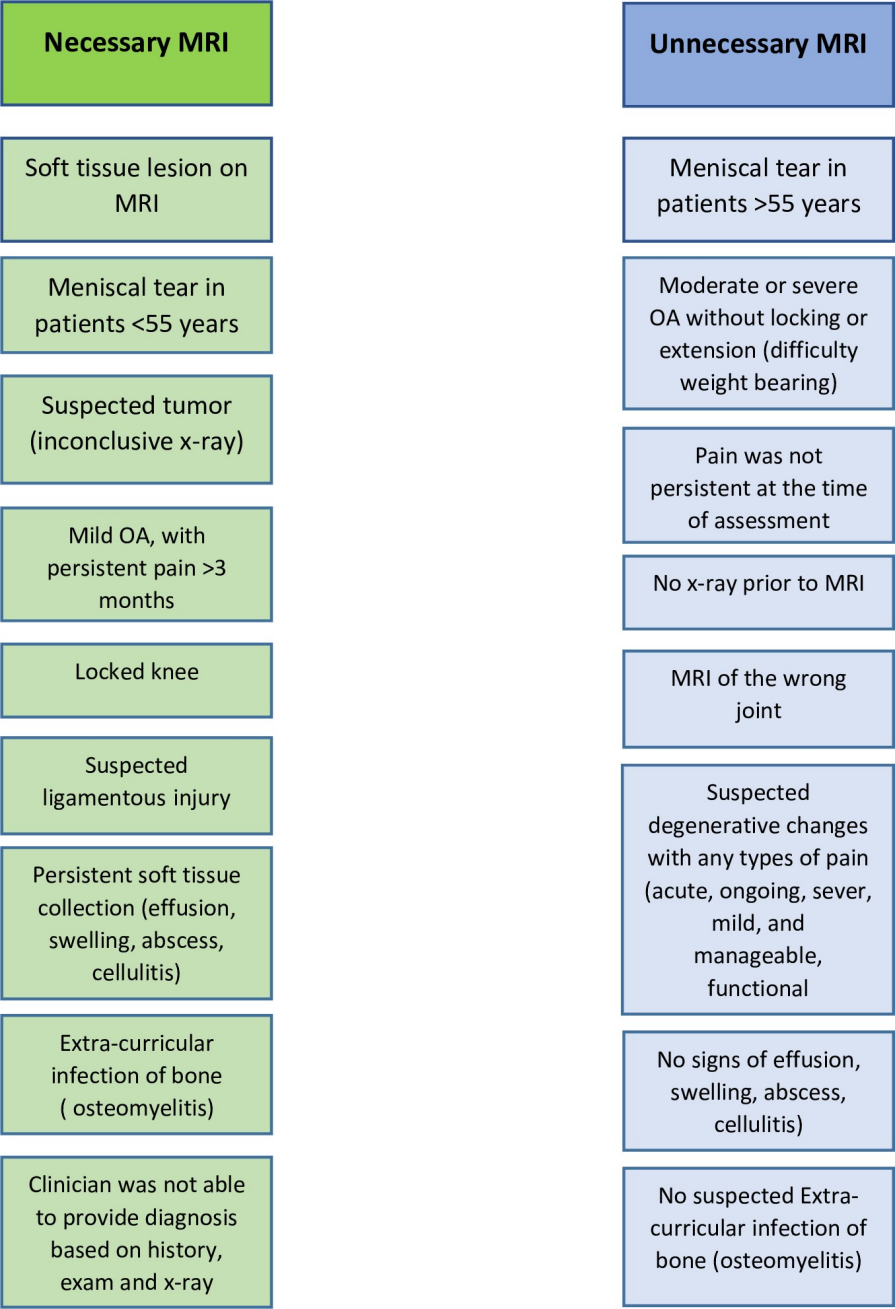
Algorithm to determine necessity of ordered knee pain MRI scans.

The principles for appropriate use of diagnostic imaging set by the Canadian Association of Radiology [15], UHN [16] and Choosing Wisely Canada [17] were used to develop decision making criteria to assist in determining the necessity of the pre-consult knee MRIs for the purposes of this study. The list of criteria used to identify unnecessary MRI scans is illustrated in Fig 1. For example, MRI scans were identified as unnecessary if the: 1) diagnosis could have been made by history, physical exam, or X-ray alone; 2) patient was asymptomatic by the time of presentation for orthopedic consultation; 3) poor or absent supportive clinical information; 4) MRI was conducted for the wrong joint; 5) no x-ray prior to the MRI scan; 6) patients with a confirmed meniscal tear are ≥55 years; 7) suspected degenerative changes with any type of pain; 8) moderate or severe osteoarthritis without a locking or extension and 9) no signs of effusion, swelling or abscess. As a final step, consultation notes were reviewed to support the decision on necessity of the MRI scans.

### Data collection

During the chart review process, patient information including age, sex, and diagnosis were recorded. The date of referral, consult, and any imaging were also noted, as well as patient disposition following consultation (i.e., conservative management, arthroscopy, or total knee arthroplasty). Some orthopedic patients, particularly those with knee pain, were first assessed at a physiotherapy assessment clinic (PAC) to determine whether they should be referred on to surgical consult. This aspect of care was documented during the chart review so that those who had attended the PAC could be analyzed separately. The data collection also included whether plain radiographs, MRIs or both types of imaging accompanied the referral, and occurrences of follow up appointments scheduled to obtain radiographs if the patient presented with an MRI only. Pathological findings from both MRI and radiographic imaging were recorded.

### Analytic approach

Descriptive analyses were conducted and summary statistics describing the study sample were expressed as number (%) for categorial variables or mean (SD) for continuous variables. Association of categorical variables based on MRI ordering as well as appropriateness of the ordered MRI scans were assessed using chi-square tests and the significance of continuous variables was assessed using independent student t test. Multivariate analysis was also done using a binominal logistic regression to predict the probability of the dependent variables based on different independent variables that are continuous and/or categorical. The strength of association or non-dependence between the variables under investigation were examined at 95% confidence intervals. Statistically significant differences were considered at p value <0.05. Data was analyzed using the Statistical Package for Social Sciences (SPSS) (SPSS; IBM Corp, Armonk, NY. Version 25; 2019).

## Results

**Fig 2:**
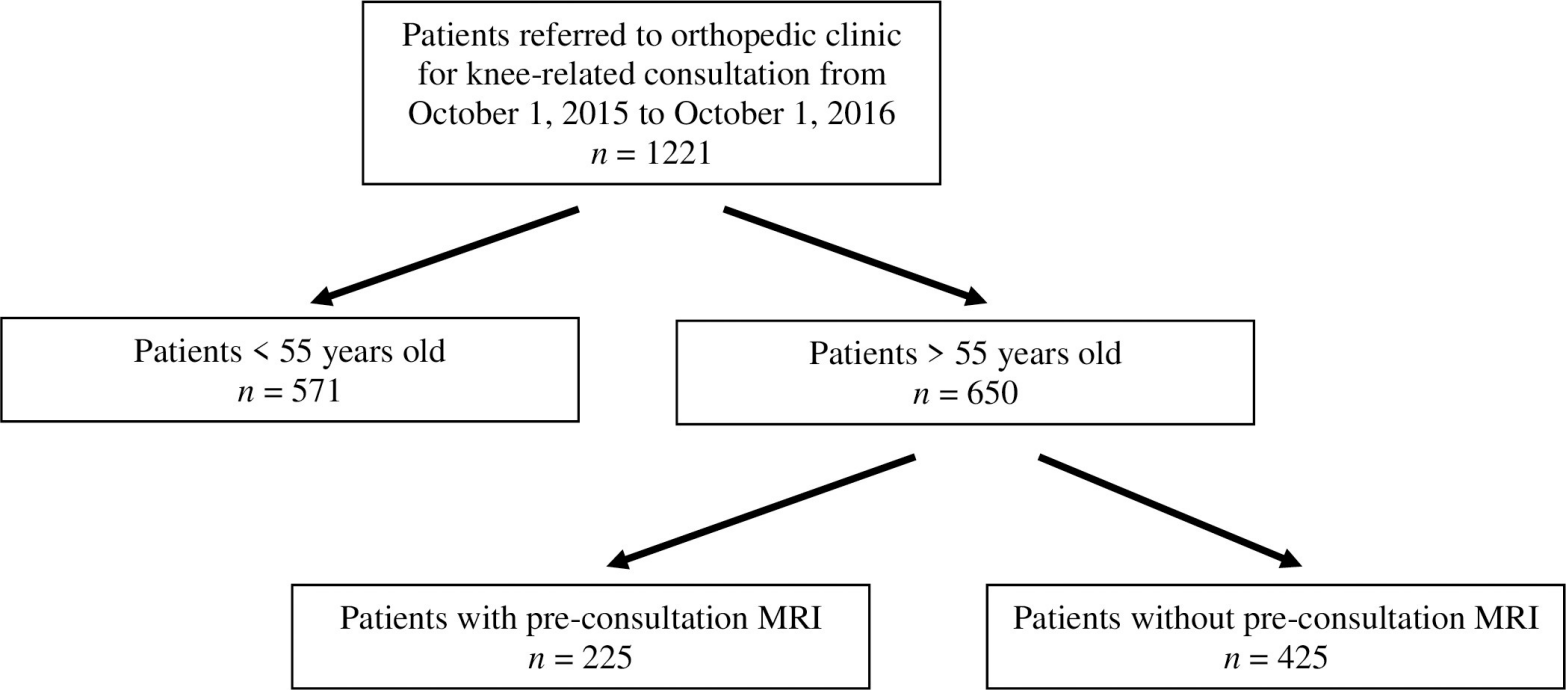
Selection process diagram.

Charts of 1221 patients with knee pain referred in the study period were identified and retrospectively reviewed. A total of 650 patients met the inclusion criteria and were included in the analysis. Of those, 225 patients (35%) had a pre-consult MRI (Fig 2).

Demographic and clinical characteristics of the overall sample are presented in Table 1. Significant differences were reported between the group with pre-consult MRI scans and those who had no MRI based on age, meniscal/ligamentous pathology as reason of referral, proportion of x-rays ordered prior to consultation, frequency of radiology ordered by the surgeon, and referral to surgeon following a PAC assessment (p<0.000). Significant differences were also detected between both groups regarding post-consult disposition outcomes (Table 1).

**Table 1:** Characteristics of knee pain MRI vs no MRI scans ordering.

	Overall	Pre-consultation MRI Group	No-MRI group	P Value
	(n = 650)	(n = 225)	(n = 425)	(MRI VS. No MRI)
**Age**				
Mean (SD)	67.79 ± 8.74	63.17 ± 7.01	70.17 ± 8.60	**P = 0.000**
**Gender**				
Females **n (%)**	368 (56.5%)	125 (55.6%)	243 (56.9%)	P = 0.707
**Time to consult (days)**				
Mean (SD)	199.96 ± 120.78	202.25 ± 131.80	198.32 ± 114.89	P = 0.707
**Reason for referral was meniscal /ligamentous pathology**				
Yes **n (%)**	131 (20%)	121 (53.8%)	10 (2.3%)	**P = 0.000**
**X-rays ordered prior to consultation**				
Yes **n (%)**	513 (78.9%)	112 (50.0%)	401 (94.2%)	**P = 0.000**
**Post-consult disposition**				
Conservative	311 (47.8%)	134 (59.6%)	177 (41.5%)	**P = 0.000**
Arthroscopy	46 (7.1%)	42 (18.6%)	4 (1.0%)	**P = 0.000**
Total Knee	294 (45.1%)	49 (21.8%)	244 (57.2%)	**P = 0.000**
Arthroplasty				
**Patients referred through MAC**				
Yes n (%)	151 (23.2%)	9 (4.0%)	142 (33.3%)	**P = 0.000**
**MRI ordered by orthopedic surgeon**				
Yes n (%)	-	-	4 (1.8%)	

Significant differences were detected between the necessary and the unnecessary pre-consultation MRI scans related to the age of patients, wait-time (in days) to consult, and the number of x-rays ordered by surgeon at the time of the consultation (p<0.000, p<0.000, and p = 0.005, respectively). For patients with an unnecessarily ordered pre-consult MRI and no x-ray, 35% required an x-ray to be ordered at the time of the surgical consult (Table 2).

**Table 2:** Characteristics of the ordered MRI pre-consultation.

	Necessary Pre-consultation MRI	Unnecessary Pre-consultation MRI	P Value
	(n = 55)	(n = 170)	
**Age**			
Mean (SD)	59.29± 4.27	64.68 ± 7.40	**P = 0.000**
**Gender**			
Females **n (%)**	24 (43.6%)	101(59.4%)	P = 0.707
**Time to consult (days)**			
Mean (SD)	141.27±108.976	222.21±132.776	**P = 0.000**
**Reason for referral was meniscal/ligament tear**			
Yes **n (%)**	34 (61.8%)	87 (51.2%)	P = 0.111
**X-rays ordered prior to consultation**			
Yes **n (%)**	27 (49.0%)	85 (50.0%)	P = 0.500
**X-ray ordered by surgeon at consultation**			
Yes **n (%)**	2 (3.9%)	30 (18.8%)	**P = 0.005**
**X-ray ordered by surgeon at consultation for patients with no prior x-ray**			
Yes **n (%)**	2 (8.3%)	26 (34.7%)	**P = 0.009**

The reported reasons for referrals with a pre-consult MRI are presented in Table 3. About half of the unnecessary MRI scans ordered for a confirmed meniscal tear for patients ≥55 years and a suspected degenerative disorder and ligament/tendon injury, 21.1% for patients with non-persistent knee joint pain and 21.8% for patients with severe or mild osteoarthritis without locking or extension.

**Table 3:** Reported diagnosis and reasons for referral of patients with an MRI.

Reason	Pre-consultation MRI	Unnecessary Pre-consultation MRI
	(N = 225)	(N = 170)
	N (%)	N (%)
**Meniscal and ligamentous pathologies**	121 (53.8%)	87 (51.1%)
**Joint pain**	46 (20.5%)	36 (21.1%)
**Osteoarthritis**	39 (17.3%)	37 (21.8%)
**Osseous pathology**	3 (1.3%)	3 (1.8%)
**Other soft-tissue pathologies**	16 (7.1%)	7 (4.2%)

Factors associated with MRI ordering are presented in Table 3. Patients referred with a reason of meniscal/ligamentous pathology were 17 times more likely to be ordered an MRI than the other patients. Patients referred to the orthopedic specialist following a PAC assessment were less likely to have an MRI ordered pre-consultation. Pre-consult x-ray was inversely associated with the ordering of MRI. The more x-rays to be ordered prior to the consultation, the less likely patients would be ordered to have an MRI by a factor of 6.99 (Table 4).

**Table 4:** Factors associated with MRI ordering- a binomial logistic regression*.

Effect**	OR	95% CI of OR	P Value
**Age**			
(continuous 55 years and older)	0.93	0.907–0.962	**P = 0.000**
**Referral was for meniscal/ligament pathology**			
Yes (vs No)	17.74	8.539–20.884	**P = 0.000**
**X-rays ordered prior to consultation**			
Yes (vs No)	0.14	0.850–0.255	**P = 0.000**
**Patients referred through MAC**			
Yes (vs No)	0.27	0.127–0.556	**P = 0.000**

^*^−2Logl = 477.153; Hosmer–Lemeshow χ^2^_8df_ = 6.568, *p* = 0.584; Nagelkerke R^2^ = 0.582. Overall percentage.86%

**Analysis adjusted for gender and time to receive consult.

A logistic regression model was computed to detect factors associated with total knee arthroplasty (TKA). It was found that patients with pre-consult MRI and patients referred with meniscal/ligamentous pathology are less likely to get TKA, while patients referred to surgeons following a PAC assessment are 4.47 times more likely to get a TKA (Table 5).

**Table 5:** Factors associated with total knee arthroplasty- a binomial logistic regression*.

Effect**	OR	95% CI of OR	P Value
**Ordered MRI pre-consult**			
Yes (vs. No)	0.424	0.258–0.698	**P = 0.001**
**Referral was for meniscal/ligament pathology**			
Yes (vs No)	0.404	0.213–0.769	**P = 0.006**
**Patients referred through MAC**			
Yes (vs No)	4.474	2.844–7.039	**P = 0.000**

^*^−2Logl = 439.743; Hosmer–Lemeshow χ^2^_8df_ = 6.280, *p* = 0.616; Nagelkerke R^2^ = 0.448. Overall percentage.67%.

** Analysis adjusted for age, gender, and time to receive consult and x-ray received prior to consultation.

## Discussion

Our study aimed to explore the prevalence and clinical necessity of the ordered MRI scans prior to receiving a surgical consult for knee pain. The results showed that 35% of patients presenting at an orthopedic clinic with knee pain had a pre-consult MRI; of those, 76% were deemed clinically unnecessary. Our findings also revealed that ordering X-ray pre-consultation was inversely associated with ordering MRI and also patients referred to the orthopedic specialists through a PAC were less likely to have an MRI ordered pre-consultation.

Our results are consistent with Huebner and colleagues’ (2019) chart review study, which reported that 84% of MRI scans ordered pre-consult were clinically inappropriate [18]. Several factors contribute to the unnecessary MRI usage in Canada. Evidence shows a variation in the appropriate MRI ordering between orthopedic surgeons and non-orthopedic clinicians [1]. Primary Care Physicians tend to order more MRIs prior to orthopedic consultation to expedite the patients’ care at the time of consult [7], or to accommodate for the long wait-time patients must endure to receive a consult [9]. Both reasons can be considered contributing factors for ordering an MRI scan when it is not indicated. This is apparent in our findings of significantly higher wait-time among patients who were ordered pre-consult MRI. Petron and colleagues (2010) reported that the orthopedic surgeons are likely to agree with the ordering of only 12% of the MRI scans they receive [7]. This underlines the critical role of a decision-support tool at the point of referral to enhance appropriate MRI ordering prior to the orthopedic consult.

Proper clinical examination and patient history complemented by radiograph images support a sound assessment [1, 7]. However, evidence shows a paucity in the frequency of radiographs ordered for knee pain patients prior to their referral to the surgeon [7, 18]. Similar to the literature [7, 18], our study shows that only half of patients who were ordered pre-consult MRI had a radiograph of the affected knee, and 35% who were ordered unnecessary pre-consult MRI with no radiographs were referred for an x-ray at the time of consult. The decision for a surgical treatment for osteoarthritis is contingent on evidence-based history, clinical examination, appropriate radiograph imaging findings rather than the presence of a meniscus tear detected with an MRI [1, 7]. Choosing Wisely Canada provides guidelines to support clinical decision-making and recommends regulating MRI ordering for degenerative meniscal tears [16].

In our study, 84% of patients considered for TKA were not ordered MRI pre-consult compared to 16% of referrals who were ordered MRI pre-consult (P<0.000). This finding highlights the limited impact of MRI scans given that they do not necessarily drive the surgeon's decision to perform a TKA. Evidence demonstrates the value of weight-bearing radiographs over MRIs in showing degenerative changes in knees [19]. Our results show that the MRI scans ordered pre-consultation were mainly for meniscal tears, suspected degenerative disorder, and ligament/tendon injury. They were primarily requested for patients ≥55 years old, with non-persistent knee pain, or signs of severe or mild osteoarthritis without locking or extension. These reasons can easily be detected by radiographs and proper clinical history and examination.

Compared to MRI scans ordered by orthopedic surgeons and non-surgical sports medicine physicians, those ordered by PCPs were less likely to have valuable findings to inform management plans [10]. Our findings underscore the need for additional education and training to close the gap in evaluating and screening musculoskeletal disorders among primary care physicians [10]. Jackson and colleagues stated that history and physical examination alone are sufficient to appropriately screen patients for many knee complaints, such as ligament injuries, and effusion, at primary care offices to make a sound decision to further refer patients to a specialist for consultation and surgical management [20]. It is critical that some orthopedic training is incorporated into frontline practices.

In any case, ordering MRI pre-consultation for the patients with significant functional joint issues for the diagnosis of meniscal tear can significantly impact patients’ management plans [2]. Evidence shows that about 20–30% of MRI results in specific knee pathologies are either false or misleading [21]. The misinforming results still maximize patients’ expectations of surgical intervention before their consultation with an orthopedic surgeon [2]. Patient’s pressure could compel a surgeon to consider arthroscopy procedures if MRI shows meniscal tear. Choosing Wisely Canada supports fewer surgical knee procedures in patients over 35 years old as the invasive arthroscopy procedures are usually not recommended for degenerative arthritis and meniscal tear [22].

Currently, patients experience a long wait time to receive an orthopedic surgical procedure in Canada [23]. Therefore, it is crucial to ensure efficient allocation of healthcare resources and wise decision making around the use of diagnostic tools that facilitate a suitable management plan and proper referral [24]. In our study, only 4% of patients referred for surgical consult following a PAC assessment had a pre-consult MRI. Upon further investigation, it was discovered that patients referred to surgeons by a PAC were four times more likely to result in a TKA. This finding is consistent with previous research that underscores the effectiveness of the skillful screening of patients referred for a pre-consult physiotherapy assessment in facilitating appropriate referrals to orthopedic surgeons and providing cost-effective care [1, 25]. Moreover, it demonstrates the value of incorporating guidelines recommendations in practice at the point of referral.

A comprehensive chart review of knee pain patients from one orthopedic clinic in Kitchener, Ontario, was included, which may not be representative of practice provincially. However, our study reviewed a relatively large sample of patient charts, which increases the statistical power of the findings. Moreover, our study had a balanced sample with an equal distribution of males to females ratio, so observations in our research setting reflect what would be observed among patients of different sex. The review of patient charts was limited to a one-year sample, so findings may not accurately reflect the reality of practice over time.

## Conclusions

Our findings revealed that many MRI scans ordered for knee pain in older patients are not clinically useful. Most of the pre-consult MRIs were found to be associated with post consultation conservative management, but some might have influenced the decision to proceed with surgical knee procedures. This highlights the necessity of following a comprehensive model of care including clinical experts and decision-support tools to facilitate evidence-based decision making within the referral practice and to reduce the amount of ordering of unnecessary MRI scans.

## Supporting information

S1 Data Retrospective chart review data. (XLSX)
